# Loss of SUMOylation drives aberrant PRC1 clustering and 3D genome rewiring independent of H3K27me3

**DOI:** 10.1093/nar/gkag851

**Published:** 2026-09-08

**Authors:** Nazli Akilli, Paul-Swann Puel, Marco Di Stefano, Fernando Muzzopappa, Lauriane Fritsch, Fabian Erdel, Daniel Jost, Thierry Cheutin, Giacomo Cavalli

**Affiliations:** Institute of Human Genetics, UMR9002, Univ Montpellier, CNRS, 34396 Cedex 5,Montpellier, France; Laboratoire de Biologie et Modélisation de la Cellule, École Normale Supérieure de Lyon, CNRS, UMR5239, Inserm U1293, Université Claude Bernard Lyon 1, 69364 Cedex 07,Lyon, France; Institute of Human Genetics, UMR9002, Univ Montpellier, CNRS, 34396 Cedex 5,Montpellier, France; MCD, Center for Integrative Biology (CBI), University of Toulouse, CNRS, 31400,Toulouse, France; Institute of Human Genetics, UMR9002, Univ Montpellier, CNRS, 34396 Cedex 5,Montpellier, France; MCD, Center for Integrative Biology (CBI), University of Toulouse, CNRS, 31400,Toulouse, France; Laboratoire de Biologie et Modélisation de la Cellule, École Normale Supérieure de Lyon, CNRS, UMR5239, Inserm U1293, Université Claude Bernard Lyon 1, 69364 Cedex 07,Lyon, France; Institute of Human Genetics, UMR9002, Univ Montpellier, CNRS, 34396 Cedex 5,Montpellier, France; Institute of Human Genetics, UMR9002, Univ Montpellier, CNRS, 34396 Cedex 5,Montpellier, France

## Abstract

Polycomb repressive complex 1 (PRC1) forms nuclear condensates that organize target chromatin domains. SUMOylation modulates PRC1 clustering, but its impact on condensate properties and 3D genome architecture remains unclear. Here, we show that depletion of small ubiquitin-like modifier (SUMO) in *Drosophila* wing imaginal discs transforms PRC1 condensates into large structures with reduced molecular dynamics. Biophysical modeling suggests that the changes in PRC1 self-interactions are responsible for the formation of large PRC1 condensates when SUMO is depleted. Interestingly, this biophysical reorganization occurs without global loss of the H3K27me3 mark. Instead, Hi-C reveals widespread rewiring of topologically associating domain (TAD) interactions. PRC1-bound TADs lose specific long-range contacts with each other while gaining ectopic interactions with active chromatin. These topological shifts correlate with gene misregulation independently of changes in Polycomb histone modifications. Our results establish SUMOylation as a critical regulator of PRC1 condensates, demonstrating that post-translational control of biomolecular condensation modulates 3D genome architecture and transcriptional output through mechanisms separable from histone mark deposition.

## Graphical Abstract

**Graphical Abstract:**
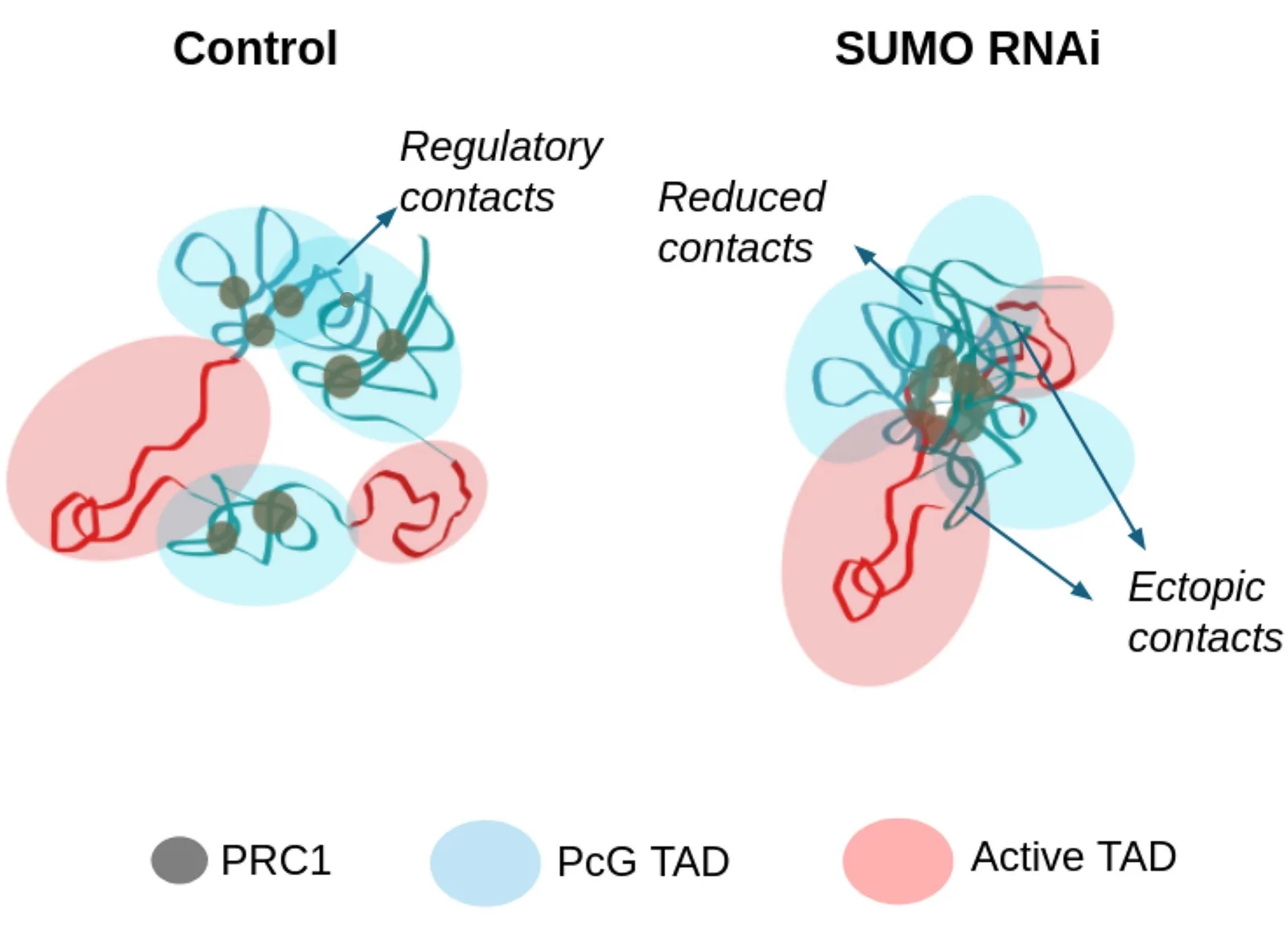

## Introduction

Polycomb group (PcG) proteins are evolutionarily conserved chromatin regulators that establish transcriptional silencing through the formation of repressive chromatin compartments. Canonical Polycomb repressive complex 1 (cPRC1), composed of core proteins Pc, Ph, Psc, and Sce, contributes to chromatin compaction, histone H2A ubiquitylation, and long-range chromatin interactions that coordinate gene repression [1]. Recent studies have revealed that PRC1 can assemble into nuclear condensates, potentially via phase separation, thereby influencing genome topology and transcriptional memory [2–6]. PRC1 condensates coincide with clustered genomic targets [7–9], highlighting a close link between condensate formation and PRC1-mediated chromatin organization [10–12].

*In vitro* studies on reconstituted nucleosome arrays have shown that both *Drosophila* and mammalian PRC1 can compact chromatin into dense aggregates that compete remodeling by Swi/Snf complexes [13]. Chromatin compaction is mediated by intrinsically disordered regions of PRC1 subunits, including Psc in *Drosophila* and CBX2 in mammalian cPRC1 [1, 14–16]. While these protein domains are central to phase separation, PRC1 is also subject to post-translational modifications such as glycosylation and SUMOylation, which can regulate the dynamics of membraneless nuclear condensates [17–19]. However, the contribution of such modifications to PRC1 phase behavior remains poorly understood.

SUMOylation involves the covalent attachment of a small ubiquitin-like modifier (SUMO) polypeptide to lysine residues of target proteins, altering their interactions and nuclear organization [20, 21]. Pc, a core subunit of PRC1, has been shown to be SUMOylated in the nucleus, and reduced SUMOylation leads to the formation of large Pc foci of ∼1 μm compared with the much smaller foci that are observed under normal conditions [18, 22]. Scm, a substoichiometric subunit of PRC1 is SUMOylated, and perturbation of SUMO levels induces Scm-dependent changes in target gene expression [17]. Despite these observations, the precise contribution of SUMOylation to PRC1 phase behavior and the resulting impact on chromatin architecture and transcriptional outcomes remains unclear.

Here, we investigate how integrity of the nuclear SUMOylation pathway modulates the phase behavior and functional output of PRC1. We hypothesized that if hypo-SUMOylated PRC1 condensates remain functionally competent, then SUMOylation may act as a molecular switch controlling PRC1 clustering and chromatin organization. Using RNAi-mediated depletion of SUMO in *Drosophila* wing discs, we examined how this global perturbation impacts PRC1 foci formation, chromatin topology, and gene expression. Our results reveal that SUMO-dependent regulation of PRC1 clustering is a key determinant of PcG-mediated chromatin organization, highlighting the profound architectural consequences of disrupting the SUMO pathway, which correlates with changes in gene expression within PcG target domains. These findings have potential implications for developmental gene regulation and the dynamic control of nuclear architecture during cell physiology and differentiation.

## Materials and methods

### Fly lines and husbandry

All *Drosophila melanogaster* stocks were maintained on standard cornmeal agar medium at 25°C under a 12 h light/dark cycle. Gene knockdown and transgene expression were achieved using the GAL4/UAS system (Brand and Perrimon, 1994) with the nub-GAL4 driver obtained from the Bloomington *Drosophila* Stock Center.

RNAi lines were sourced from the Vienna *Drosophila* RNAi Center and the Transgenic RNAi Project (TRiP; smt3 RNAi, stock JF02869). The following genotypes were used in this study:

- w [1118]; P{w [+mC]=UAS-Dcr-2.D}1; P{w [+mW.hs]=GawB}nubbin-AC-62 (nub-GAL4 driver line)
- PcGFP/CyO; Smt3RNAi/TM6B (SUMO RNAi)
- Actin5C-PcGFP/FM7 (overexpression of Pc-GFP)

Crosses were performed at 25°C, and F1 progeny were used for downstream experiments. All fly lines used in this work are the same as the ones used in Gonzales *et al*., 2014.

### Immunostaining

Homemade antibodies were pre-cleaned prior to the staining procedure. For pre-cleaning of antibodies against PRC1 components, at least 20 larvae were dissected, retaining imaginal discs but removing fat tissue. Carcasses were transferred to 1.5-ml microcentrifuge tubes containing 750 µl phosphate buffered saline (PBS), and fixation was performed by adding 250 µl 16% paraformaldehyde (4% final) for 20 min at room temperature on a rotating wheel. Samples were washed three times with PBS, then permeabilized in 0.5% Triton X-100 in PBS (PBTr) for 1 h at room temperature with gentle rotation; the PBTr solution was replaced 2–3 times during this step. Tissues were subsequently blocked for 1 h in 3% bovine serum albumin (BSA) prepared in 0.1% PBTr at room temperature.

Primary antibodies were diluted 1:500 (Ph and Pc antibodies) in PBTr containing 1% BSA. Carcasses were incubated with the antibody solution for 2 h at room temperature, after which the antibody solutions were recovered and used for staining newly dissected samples.

For staining, newly dissected larvae were processed as above (4% paraformaldehyde fixation for 20 min, 0.5% Triton X-100 permeabilization for 1 h, and blocking in 3% BSA for 1 h) and then incubated overnight at 4°C with the pre-cleaned antibody solutions. The following day, tissues were washed 4–6 times with 0.1% PBTr at room temperature, and then incubated with secondary antibodies (1:200 dilution in PBTr containing 1% BSA) for 2 h at room temperature. Samples were washed at least three times for 15 min each in 0.1% PBTr.

Nuclei were counterstained with 1 µg ml^−1^ DAPI in 0.1% PBTr for 20 min at room temperature, followed by three 15-min washes in 0.1% PBTr and three quick PBS washes without detergent. Wing discs were then dissected from carcasses in PBS and mounted in Vectashield (Vector Laboratories).

### RNA preparation for RNA-sequencing

Third-instar larvae were dissected in Schneider medium. For SUMO RNAi and for respective control, only male larvae were dissected. Total RNA was extracted using TRIzol reagent, and extracted RNA was purified using RNA Clean & Concentrator kit (Zymo Research, #R1015). The sequencing was designed for messenger RNAs; thus, poly-A RNA enrichment, library preparation, and Illumina sequencing were performed by BGI. 6 GB of data were produced for each sample by BGI using paired-end 150-bp sequencing.

### RNA-seq analysis

RNA-seq samples were aligned using STAR aligner (v2.7) [45]. Subread (v2.0.6) [46] was used to create the count table with the command *featureCounts -p –countReadPairs -s 2*. This count table served as the input for DESeq2 (v1.40.2) [47]. The volcano plots were obtained using the R library EnhancedVolcano (v1.20.0).

### CUT&RUN experiments

CUT&RUN experiments were performed as described by Kami Ahmad in https://www.protocols.io/ (10.17504/protocols.io.umfeu3n) with minor modifications. We dissected 20 wing discs for histone marks and 40 WDs for indirect DNA-binding proteins in Schneider medium, centrifuged them for 3 min at 2000 × *g*, and washed them twice with wash+ buffer before adding concanavalin A-coated beads. For experiments involving Pc, we included a 5-min crosslinking step with 0.1% formaldehyde on the wheel at room temperature. MNase digestion (pAG-MNase Enzyme from Cell Signaling) was performed for 30 min at 4°C on shaker. After Proteinase K digestion, DNA was recovered using SPRIselect beads and eluted in 15 μl of Tris 10 mM. DNA libraries for sequencing were prepared using the NEBNext Ultra II DNA Library Prep Kit for Illumina. Sequencing (paired-end sequencing 150 bp, roughly 2 Gb per sample) was performed by Novogene (https://en.novogene.com/). The following antibodies were used: H3K27me3 (1:100, Active Motif, catalogue no. 39155), H2AK118Ub (1:100, Cell Signaling, catalogue no. 8240), H3K27ac (1:100, Active Motif, catalogue no. 39135), H3K9me3 (1:100, Active Motif, catalogue no. 39161), Pc (home made by G. Cavalli lab). All experiments were performed in biological duplicates.

### CUT&RUN analysis

CUT&RUN samples for H3K27me3, Pc, H2Aub118, H3K27ac, and IgG in Control and SUMO RNAi imaginal discs were aligned using Bowtie2 (v2.4.2) with the options *-p 8 –local –very-sensitive-local –no-unal –no-mixed –no-discordant –phred33 -I 10 -X 700*. Low-quality reads were filtered with samtools (v1.9) using the command “samtools view -F 4 -h -q 30.” The resulting BAM files were sorted, indexed, and de-duplicated using sambamba (v1.0.0) with commands *sambamba sort* and *sambamba markdup –remove-duplicates* respectively. To generate normalized BigWig files, the bamCoverage command from deepTools2 (v3.5.5) was used with parameters *–normalizeUsing RPKM –ignoreDuplicates. macs3* (v3.0.0b1) was used to call peaks on each replicate with commands macs callpeak -t -f BAMPE -g 142 573 017 -q 0.01 -c ${controlBam} for Pc and H3K27ac and macs3 callpeak -t ${sortedDedupBamPerReplicate} -c ${controlBam} -f BAMPE -g 142 573 017 -q 0.01 –broad –broad-cutoff 0.01 for H3K27me3 and H2Aub118. For each condition, the peaks per target were defined as the intersection between the peaks called in each of the replicates using the command *bedtools intersect* (v2.31.1). R library DiffBind (v3.10.1 on R v4.3.3) was used to find differential binding regions on the union of the peaks obtained for the SUMO RNAi and Control conditions. To define chromatin states in the Control condition, the C&R for H3K9me3 mark in Wing imaginal disc of *D. melanogaster* was obtained from the GEO entry GSE255496 (GSM8073294 and GSM8073295) together with its IgG samples (GSM8073298). ChromHMM analysis was applied by first binarizing the signal in the replicate-merged .bam files of H3K27me3, Pc, H3K27ac, and H3K9me3 using IgG as a control track (command: *java -mx40G -jar ChromHMM.jar BinarizeBam -b 200 -p 0.0001./${bamFilesDir}/ cellmarkfiletable.tsv./${binarizedBamFilesDir}*) and next by defining a four-state model based on the binarized signal (command: *java -mx40G -jar ChromHMM.jar LearnModel -b 200./${binarizedBamFilesDir}./${modelDir} 4 dm6*). The resulting four ChromHMM emissions were assigned to chromatin states: Polycomb (blue) chromatin domains enriched by H3K27me3, Active (red) chromatin enriched by H3K27ac, Heterochromatin (green) enriched by H3K9me3, and Null (black) chromatin not enriched in any features used for the analysis.

### Hi-C experiments

Hi-C was performed using the EpiTech Hi-C Kit (Qiagen) according to the manufacturer’s instructions with minor modifications.

Sixty wing discs were dissected in Schneider’s medium and transferred to 200 µl HBSS containing 10.8 µl 37% formaldehyde (2% final). Samples were homogenized using a Biomasher II (Funakoshi, 320103) and fixed for 20 min at room temperature. Crosslinking was quenched for 5 min with 100 µl 3 M Tris–Cl (pH 7.5), followed by addition of 650 µl HBSS and gentle inversion. The collagenase treatment from the manufacturer’s protocol was omitted, as it reduced DNA yield. Samples were centrifuged (800 × *g*, 5 min, 4°C), and 250 µl QIAseq Beads (equilibrated to room temperature) were added to the remaining ∼250 µl containing nuclei. After 10 min at room temperature, beads were magnetically separated and washed sequentially with 50 µl ice-cold PBS, 150 µl cold RNase-free water, and 50 µl cold Buffer C1. The mixture was incubated on ice for 10 min and then at room temperature for an additional 10 min before a final wash with 500 µl cold RNase-free water.

For chromatin digestion, beads were resuspended in 40 µl Hi-C Digestion Solution and incubated at 65°C for 10 min, followed by cooling on ice. Subsequently, 4.4 µl 10% Triton X-100 and 4 µl Hi-C Digestion Enzyme were added, and samples were incubated at 37°C for 2 h (600 rpm) and then at 65°C for 20 min. Samples were placed on ice and could be frozen at this stage.

End labeling was performed by sequential addition of 6 µl Hi-C End Labeling Mix and 1 µl Hi-C End Labeling Enzyme, with gentle mixing and incubation at 37°C for 30 min. For ligation, 350 µl Hi-C Ligation Solution was added, and samples were gently inverted and incubated at 16°C for 2 h. Samples were cooled on ice before proceeding or frozen for later use.

For de-crosslinking, 10 µl Proteinase K was added, and samples were incubated at 56°C for 30 min and at 80°C for 90 min, then cooled to room temperature. DNA was precipitated by adding 40 µl 3 M sodium acetate (pH 5.2) and 280 µl isopropanol, vortexed briefly, and applied (including beads) to a MinElute® column (Qiagen). The column was washed with 0.75 ml Buffer PE, centrifuged (17 900 × *g*, 1 min), and eluted with 35 µl Buffer EB pre-warmed to 65°C.

Recovered DNA was diluted to 130 µl and sonicated for 90 s using a Covaris S220 (140 W peak power, 10% duty factor, 200 cycles per burst). Sonicated DNA was purified by adding four volumes of Buffer SB1, loading onto a MinElute column, washing twice with 700 µl 80% ethanol, and eluting with 50 µl Buffer EB pre-warmed to 65°C.

### Hi-C analysis methods

Raw data from Hi-C sequencing were processed using the “scHiC2” pipeline. Valid interactions were stored in a database using the “misha” R package (https://github.com/msauria/misha-package). Extracting the valid interactions from the *misha* database, the “shaman” R package (https://bitbucket.org/tanaylab/shaman) was used for computing the Hi-C expected models and Hi-C scores with parameters *k* = 250 and *k_exp* = 500. Specifically, Hi-C scores quantify the contact enrichment (positive values) or depletion (negative values) of each bin of the map with respect to a statistical model used to evaluate the expected number of counts. To generate this expected model, we randomized the observed Hi-C contacts using a Markov chain Monte Carlo-like approach per chromosome. Shuffling was conducted such that the marginal coverage and decay of the number of observed contacts with the genomic distance were preserved, but any features of genome organization [e.g. topologically associating domains (TADs) or loops] were not. These expected maps were generated for each biological replicate separately and contained twice the number of observed *cis* contacts. Next, the score for each contact in the observed contact matrix was calculated using the *k*-nearest neighbors strategy. In brief, the distributions of two-dimensional Euclidean distances between the observed contact and its nearest *k_exp* neighbors in the pooled observed and pooled expected (per cell type) data were compared, using Kolmogorov–Smirnov *D* statistics to visualize positive (higher density in observed data) and negative (lower density in observed data) enrichments. These *D* scores were then used for visualization (using a scale from −100 to +100) and are referred to as Hi-C scores in the text. Accordingly, the color scale of the Hi-C scores comprises both positive and negative values. *.mcool* were obtained using *cooler* [48] (v0.10.2) and *cooltools* [49] (v0.7.0) and normalized via the Iterative Correction and Eigenvector decomposition algorithm (ICE) with default parameters (command “*cooler zoomify -r 1000,2000,5000,10000,15000,20000,40000,50000,100000 file.cool -o file.mcool –balance*”). The *Z*-score differential map at 10-kb resolution shown in Fig. 4a was obtained as follows: first, by computing the pixel-by-pixel matrix of differences of the SUMO RNAi and Control ICE-normalized maps in the plotted region (*D_ij_*), next by computing the mean and standard deviation of all the entries (*mean* and *std.dev*.), and, finally, computing the matrix of *Z*-scores (*Z_ij_=(D_ij_-mean)/std.dev*.). Positive *Z*-scores indicate more contact propensity in SUMO RNAi, while negative *Z*-scores means more contact propensity in Control. The physical domains (TADs) were called in the Control condition using the TopDom algorithm (v0.10.1) [50] as in the HiCExperiment R-package (v1.4.0) [48] using a window size parameter of 25 kb on the *.mcool* files at 5 kb resolution. Each of the 5-kb TAD borders was then refined by moving it at the 1-kb point of minimum insulation score as computed using *cooler* with a *window* parameter of 25 kb in the −5/5 kb interval around the TopDom detected border. The distributions of TAD overlaps in Fig. 4b between any two TAD sets in condition (or replicate) A and B were computed by taking each of the TADs in partition A, T_A_, computing the overlap (defined as the percentage of bins in common) with each TAD in partition B, and selecting per each T_A_ the maximum overlap. Each distribution has a number of points n_A_ equal to the number of T_A_s. Box plots show the median (central line), the 75th and 25th percentiles (box limits) and 1.5 × IQR (whiskers).

### Image acquisition

For confocal and the half-FRAP experiments, an LSM980 microscope was used. For half-FRAP, time series were recorded every 150, 200, 300, 350 or 500 ms. Bleaching was performed at the third time point. The pixel size was 30 nm for the images. The pinhole was increased to 150 µm. AiryScan microscopy was performed using a LSM 980 microscope (Carl Zeiss Microscopy, Iena, Germany) with a AiryScan2 detector and a 63 × PlanApo objective having a numerical aperture (N.A.) of 1.4. Pc-GFP was excited with a 488 nm laser diode, and we used the mode AiryScan SR to collect images with an optimal pixel size of 42 nm in *x* and *y* and 170 nm in *z*. Processing of raw images to produce AiryScan images was done with the Zen software controlling the microscope, with a Wiener filter automatically adjusted by the software.

### Image analysis

**Figure 1.**
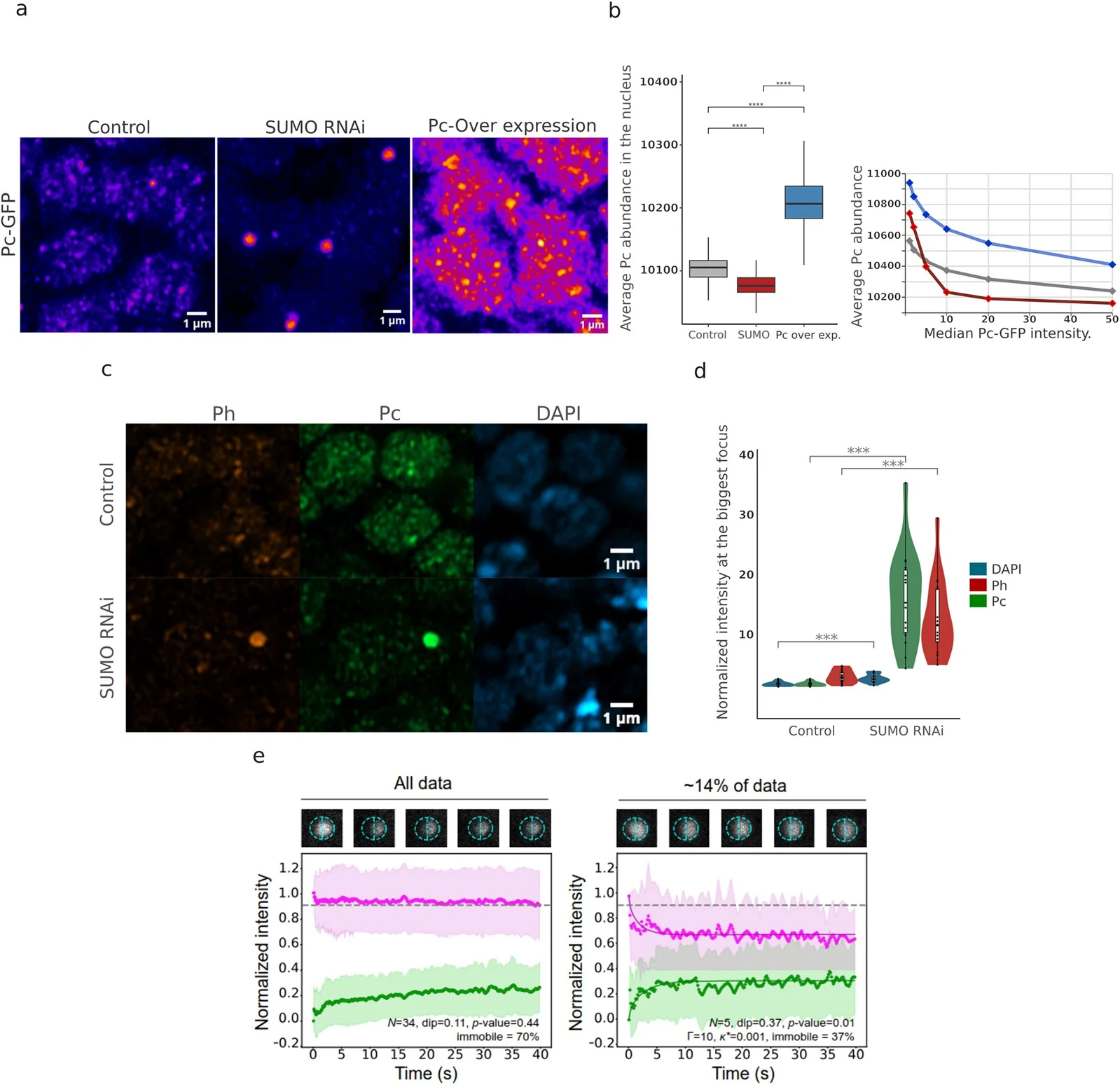
SUMO RNAi leads to large PRC1 foci formation. (**a**) Two-micrometer-thick projections showing the nuclear distribution of Pc-GFP in wing discs of control, SUMO-KD, and overexpressing Pc-GFP flies. The pseudo-color images show that few Pc-GFP foci become very intense in the SUMO-KD, while the intensity of all foci increases when Pc-GFP is overexpressed. Scale bar: 1 µm. (**b**) Box plots comparing median Pc-GFP intensities in cell nuclei of control, SUMO-KD, and overexpressing Pc-GFP (blue, *n* = 84 110) wing discs (left). Compared to control wing discs, the intensity of nucleoplasmic Pc-GFP significantly decreases in SUMO-KD, whereas it strongly increases when Pc-GFP is overexpressed. Cumulative histograms comparing the intensity of Pc-GFP foci in control (gray, *n* = 77 699), SUMO-KD (red, *n* = 3941), and Pc-GFP-overexpressing (blue, *n* = 84 110) wing discs (right). Pc-GFP foci are ranked in descending order of intensity, and the points corresponding to the first, second, fifth, 10th, 20th, and 50th percentiles are shown. (**c**) Airyscan images of immunostaining against Ph, Pc, and DAPI staining, in control sample (top) and SUMO RNAi sample (bottom). (**d**) Quantification of the immunostaining in control (*n* = 21) and SUMO (*n* = 17) RNAi samples Pc (green), Ph (red), DAPI (blue). Normalized intensity indicates the intensity of the signal within the largest focus in the nucleus, divided by the total intensity inside the nucleus for the respective signal. (**e**) Average MOCHA-FRAP curve of all the analyzed foci (*n* = 34, dip = 0.11, *P*-value = .44). The dip depth with a *P*-value above .05 indicates that the majority of the PRC1 foci do not show liquid-like behavior with an interfacial barrier (left). MOCHA-FRAP curves of a subpopulation of 5 foci (that are included in the average curve shown on the left) that exhibit liquid-like behavior with an interfacial barrier (*n* = 5, dip = 0.37, *P*-value = .01) (right). Solid lines represent the theoretical recovery for an internally mixed condensates with a Pc-GFP enrichment of Γ=10 and a vanishing permeability of κ* = 0.001. In both panels, the curve in purple indicates the change in intensity of the non-bleached half of the focus, and the curve in green indicates the recovery of the intensity of the bleached half of the focus. The fraction of immobile molecules in the focus can be detected by the final distance between the purple and the green curves, as indicated in the panel. In panels (b) and (d), boxplots show median (central line), Q1 = 25th and Q3 = 75th percentiles (box limits), and Q1 + 1.5 × IQR to Q3 + 1.5 × IQR (whiskers), where IQR is the interquartile range. Outliers are not shown.

To characterize Pc foci inside cell nuclei (Fig. 1), we quantified 3D images acquired in Airy Scan microscopy by using the software Imaris 9.8.0 (Oxford Instruments, UK). We applied the spots option of the Imaris software to identify nuclear foci and measure their Intensity. To estimate the intensity of Pc-GFP inside the nucleoplasm, we used the median intensity of Pc-GFP inside cell nuclei.

### Polymer model

PRC1 complexes and chromatin are modeled following the framework developed in previous work [51]. Briefly, PRC1 molecules are represented as a lattice gas with ${{N}_P}$ particles, i.e. as beads of effective size $20\ \mathrm{ nm}$ residing on the vertices of a face-centered cubic lattice [52]. Similarly, chromatin is described as a semi-flexible, self-avoiding polymer chain composed of ${{N}_C}$ monomers evolving on the same lattice as the PRC1 molecules. Each monomer encompasses $1\ \mathrm{ kbp}$ of chromatin and can be in one of two epigenetic states: H3K27me3-marked (Polycomb-regulated regions) or H3K27me3-unmarked (the rest of the chromatin). The Hamiltonian H of the system describing its dynamics is composed of four terms: $H = {{H}_{\mathrm{bend}}} + {{H}_{P - P}} + {{H}_{P - H}} + {{H}_{SH}}$.

${{H}_{\mathrm{bend}}} = \mathop \sum \nolimits_{i = 2}^{{{N}_C} - 1} \kappa ( {1 - \mathrm{ cos}{{\theta }_i}} )$ accounts for the bending rigidity of the chromatin where ${{\theta }_i}$ is the angle between monomers $( {i - 1,i,i + 1} )$ and $\kappa = 3.2\ {{k}_B}T$ is the bending modulus of the polymer chain, corresponding to a Kuhn length of $100\ \mathrm{ nm}$ [53, 54].

${{H}_{P - P}} = - \frac{{{{E}_{P - P}}}}{2}\mathop \sum \nolimits_{i\in \mathcal{P}}^{} \mathop \sum \nolimits_{l\in \mathcal{V}( i )}^{} {{\sigma }_l}$ accounts for the self-attractions between PRC1 molecules where ${{{\boldsymbol{E}}}_{{\boldsymbol{P}} - {\boldsymbol{P}}}}$ is the strength of self-attraction, $\mathcal{P}$ is the ensemble of PRC1s, $\mathcal{V}( i )$ are the lattice sites that are nearest-neighbor of the one where PRC1 *i* is seated, and ${{\sigma }_l} = 1$ if a PRC1 is on lattice site *l* (${{\sigma }_l} = 0$ otherwise).

${{H}_{P - H}} = - {{E}_{P - H}}\mathop \sum \nolimits_{i\in \mathcal{P}}^{} \mathop \sum \nolimits_{l\in \mathcal{V}( i )}^{} {{\kappa }_l}$ accounts for the cross-interactions between PRC1 molecules and H3K27-marked monomers where ${{E}_{P - H}}$ is the strength of cross-interaction and ${{\kappa }_l} = 1$ if an H3K27-marked monomer is on lattice site *l* (${{\kappa }_l} = 0$ otherwise).

${{H}_{SH}} = + {{E}_{SH}}\mathop \sum \nolimits_{i\in \mathcal{P}}^{} {{\sigma }_i}{{\pi }_i}$ accounts for steric hindrance interactions between PRC1 molecules and any monomers where ${{E}_{SH}}$ is the strength of steric hindrance and ${{\pi }_i}$ is the number of monomer on the lattice site *i*.

The lattice is made of *N* vertices, with periodic boundary conditions. Each vertex has 12 nearest neighbors and can contain at most one PRC1 molecule and 2 chromatin monomers. Note that double occupancy by monomers is allowed if and only if monomers are consecutive along the chain, accounting for bond fluctuations while ensuring correct self-avoidance [54].

### Monte Carlo simulation

Simulations were performed starting from random, unknotted initial configurations for the chromatin chain and a uniform random distribution for PRC1 molecules. The system is evolved via a kinetic Monte Carlo scheme, as detailed in previous work [51]. Briefly, each Monte Carlo step (MCS) consists of ${{N}_C}$ monomer trial moves and ${{N}_P}$ PRC1 trial moves. During a trial move, a PRC1 molecule or a monomer is randomly selected and an attempt to move it to one of randomly chosen nearest neighbor vertex is attempted. The new configuration is accepted via the Metropolis criterion based on the energy difference between the trial and current configurations.

For each investigated parameter set, we simulated ${{N}_{\mathrm{traj}}} = 40$ stochastic trajectories, each composed of a first warm-up of 10^6^ MCS in absence of self- and cross-interactions to relax the system, followed by 10^7^ MCS with the full Hamiltonian, where snapshots were extracted every 10^5^ MCS.

We simulated chromosome arm 3R (${{N}_C} = 29\,184$ monomers) including *2859* H3K27-marked monomers distributed in several domains across the polymer based on ChIP-seq data in the wing disk. The ratio ${{R}_{P/H}}$ between ${{N}_P}$ and the number of H3K27-marked monomer was varied from *50% * to *100% *, ${{E}_{P - P}}$ from *0.2* to $2.2\ {{k}_B}T$, ${{E}_{P - H}}$ from *0.8* to $1.2\ {{k}_B}T$ and ${{E}_{SH}}$ from *8* to $10\ {{k}_B}T$. The set of parameters (${{E}_{P - P}} = 1\ {{k}_B}T$, ${{E}_{P - h}} = 0.8\ {{k}_B}T$, ${{E}_{SH}} = 8\ {{k}_B}T$ and ${{R}_{P/H}} = 50\% $) is representative of the wild-type condition, capturing the strength of typical interactions of architectural proteins having phase-separation properties [2, 51, 55] and the typical *in vivo* concentration of PRC1 [56]. Our results are not qualitatively dependent on this choice.

### Data analysis of the simulations

For each snapshot, PRC1 foci were defined similarly as in previous work [51]. Briefly, for each PRC1 molecule *i*, we defined its local PRC1 density ${{\eta }_i} = \frac{1}{{12}}\mathop \sum \nolimits_{l\in \mathcal{V}( i )}^{} {{\sigma }_l}$ and its local H3K27-marked monomer occupancy ratio ${{\zeta }_i} = \frac{1}{{26}}\mathop \sum \nolimits_{l\in \mathcal{V}( i )}^{} {{\kappa }_l}$. The ensemble of PRC1s belonging to a condensate is defined as $\mathcal{D}\ = \ \{ {{{\eta }_i} + {{\zeta }_i} \ge 0.5,\ i\in \mathcal{P}} \}$.

The connected components of $\mathcal{D}$ are the foci. The volume of each focus is defined as the number of PRC1s inside it. The PRC1 free nucleoplasmic fraction is computed as $( {{{N}_P} - \mathrm{Card}( \mathcal{D} )} )/{{N}_P}$. The PRC1 free nucleoplasmic fraction, the number of foci, and the distribution of foci volume were averaged over the 10 last frames of each simulation.

## Results

### Loss of SUMOylation transforms PRC1 condensate morphology and dynamics

To decipher the mechanism of Polycomb foci formation and address the role of PRC1 phase separation *in vivo*, we took advantage of the previously reported observation that Pc-GFP accumulates in large, intense nuclear foci upon SUMO inactivation [18]. We compared the formation of Polycomb foci in fly lines that constitutively expressed Pc-GFP where we either induced a SUMO-KD by down-regulating *smt3*, the only SUMO factor in *D. melanogaster*, or overexpressed Pc-GFP in the pouch of wing imaginal discs. Compared to control discs, AiryScan imaging confirmed that SUMO-KD induces the formation of few, large (frequently a single one), intense Polycomb foci per cell nucleus. On the other hand, overexpression of Pc-GFP increases the intensity of all Polycomb foci (Fig. 1a) without merging them into fewer and larger ones. This result indicates that the formation of large Polycomb foci in SUMO-KD does not simply result from an increase in the nuclear concentration of Pc-GFP, but suggests that SUMO-KD specifically affects the condensation properties of Pc-GFP.

Since phase behavior is often concentration-dependent [23, 24], we asked whether SUMOylation affects Pc protein abundance. To estimate the free amount of nuclear Pc-GFP,i.e. the fraction of Pc-GFP not located in Polycomb foci, we measured the median intensity of Pc-GFP in the cell nuclei of control, SUMO-KD, and Pc-GFP-overexpressing wing discs. In addition to the formation of few large, intense Polycomb foci, SUMO-KD also significantly reduced the level of Pc-GFP outside foci, compared to control conditions (Fig. 1b), suggesting that SUMO inactivation favours the association of Pc-GFP with foci. On the other hand, Pc-GFP overexpression increases the level of free Pc-GFP in cell nuclei (Fig. 1b). To quantify the accumulation of Pc-GFP in Polycomb foci, we computed local maxima to detect Pc-GFP foci and then ranked their intensity in descending order to compare the control, SUMO-KD, and overexpression conditions. The intensity of Pc-GFP in all nuclear foci is higher than that observed in control wing discs when Pc-GFP is overexpressed. Conversely, only a few Pc-GFP foci (~5%) show a higher intensity in SUMO-KD wing discs than in control discs. The remaining foci show a lower intensity in SUMO-KD, suggesting that most of the Pc-GFP accumulates in a few intense foci (Fig. 1a–c). Thus, SUMO loss alters condensate properties rather than simply stabilizing Pc protein. To verify that the localization of Pc-GFP reflects the nuclear distribution of PRC1, we performed immunolabeling experiments using the PRC1 subunit Ph and counterstained them with DAPI. Super-resolution Airyscan imaging of both control and SUMO-KD wing discs shows a strong co-localization between Ph and Pc subunit in both conditions, demonstrating that the large, intense Polycomb foci observed upon SUMO-KD correspond to PRC1 structures (Fig. 1c). In addition, the DAPI signal also co-localizes with the large PRC1 foci upon SUMO-KD, confirming their chromatin-associated nature rather than off-chromatin aggregation (Fig. 1c and d; Supplementary Fig. S1b and c).

PRC1 condensates have been proposed to form via liquid–liquid phase separation [2–6, 10, 15, 16, 25, 26]. To specifically test this point, we next asked whether SUMOylation regulates Pc-GFP phase behavior by performing calibrated half-FRAP (MOCHA-FRAP) [27]. The purpose of this assay is to distinguish between liquid-like condensates with a significant interface barrier (characterized by a dip in the unbleached half curve due to intermixing) and condensates where exchange with the nucleoplasm dominates, reflecting assemblies formed by simple chromatin binding or by polymer–polymer phase separation. Analysis of PRC1 foci revealed that the large majority lacked the characteristic dip of liquid-like condensates (Fig. 1e), suggesting restricted internal dynamics and a shift toward a gel-like or solid-like state upon SUMO loss. Consistent with this interpretation, FRAP analysis revealed a mobile fraction of only ∼30% in PRC1 condensates (Fig. 1e), indicative of a stable scaffold with limited molecular exchange. However, ∼14% of foci did show a small dip, suggesting that a sub-population of large Pc-GFP foci do have liquid-like properties upon SUMO KD. These experiments could only be performed on the wing discs where SUMO RNAi is induced and not in control wing discs because Pc-GFP foci are too small to be half-bleached in this tissue. However, upon SUMO depletion PRC1 condensates transform into clearly larger, morphologically distinct structures that are characterized by a lower internal dynamics than controls [18] and by the absence of the surface tension that would be expected from a phase-separated liquid structure (Fig. 1e). The observation of liquid behavior in few cases suggests the possibility of a dynamic regulation of the properties of PRC1 foci during the accumulation of Pc-GFP upon SUMO KD.

### Changes in PRC1 self-interactions can explain the aberrant clustering behavior of PRC1 upon SUMO RNAi

**Figure 2.**
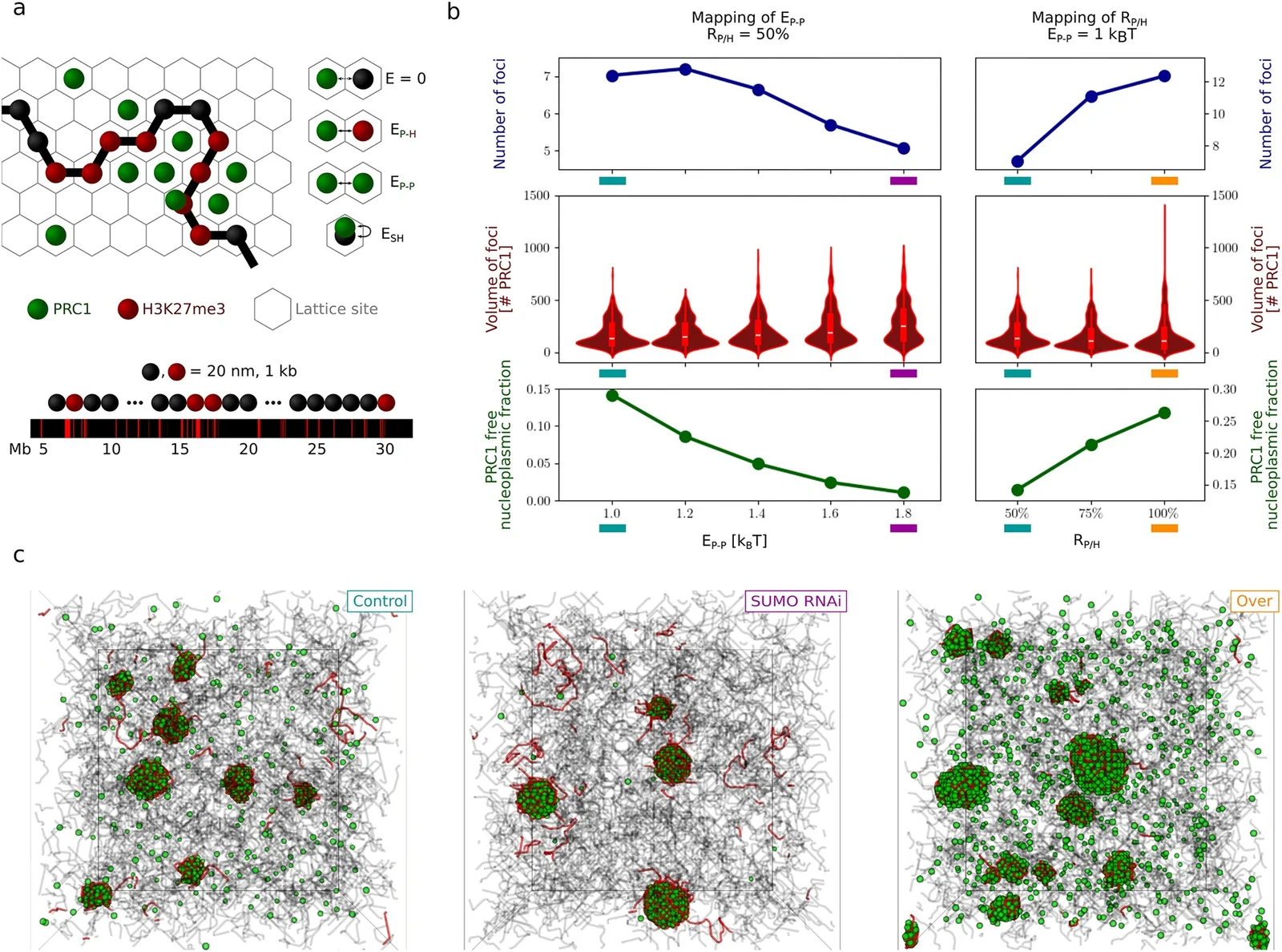
PRC1 self-interaction recapitulates the effect of SUMO RNAi. (**a**) Scheme of the chromatin and PRC1 simulation. Self-interacting PRC1 complexes, (in green, ${{E}_{P - P}}$) exhibit interaction for H3K27me3-marked monomers (${{E}_{P - H}}$, in red). Steric hindrance (${{E}_{SH}}$) between PRC1 and monomer (both black and red) is taken into account. The 3R chromosome model is modeled from ~5 to 30 Mbp with H3K27me3 profiles approximating the size and location of PcG region in chromosome 3R of *Drosophila*. (**b**) Dependence of the number of foci, their volume distributions (in number of PRC1), and of the PRC1 free nucleoplasmic fraction on the PRC1 energy of self-interaction ${{E}_{P - P}}$ (left) as well as on the ratio between PRC1 complexes and H3K27me3 marked monomers ${{R}_{P/H}}$(right). The ratio ${{R}_{P/H}}$ is first fixed to 50% for the mapping of ${{E}_{P - P}}$. The energy ${{E}_{P - P}}$ is fixed to 1 k_B_T for the mapping of ${{R}_{P/H}}$. Wild-type-like parameters are marked in teal. Purplely and orangely-marked parameters are representative of the SUMO RNAi and the over-expressed conditions, respectively. (**c**) Example of the final frame for each parameter set described in panel (b). In panel (b), boxplots show median (central line), Q1 = 25th and Q3 = 75th percentiles (box limits), and Q1 + 1.5 × IQR to Q3 + 1.5 × IQR (whiskers), where IQR is the interquartile range. Outliers are not shown.

To investigate how SUMO depletion can lead to the formation of large PRC1 condensates, we developed a generic biophysical framework coupling the interacting properties of PRC1 molecules with chromatin mechanics (see “Materials and methods” section). We considered a coarse-grained polymer representation of *Drosophila* chromosome 3R at 1 kbp resolution (29 Mbp), featuring multiple H3K27me3-marked domains that can specifically interact with ${{N}_P}$ diffusible self-attracting PRC1 molecules (Fig. 2a). Using this framework, we systematically investigated multiple parameter sets to characterize the formation of PRC1 foci and its impact on chromosome organization, in control and perturbed conditions (Fig. 2b and c; Supplementary Fig. S2). We assumed that the observation of large Polycomb foci does not constitute evidence of phase separation if their formation relies solely on the direct binding of PRC1 to chromatin. Conversely, the requirement for homotypic interaction between PRC1 molecules to explain their accumulation in the foci observed in SUMO-KD would indicate that phase separation plays an important role, since the formation of the condensate would not be explained by the binding of PRC1 to chromatin alone.

In particular, we tested the impact of the strengths of homotypic attractions between PRC1 complexes (${{E}_{P - P}}$) and of heterotypic interactions between PRC1 and H3K27me3-marked loci (${{E}_{P - H}}$) for different concentrations of PRC1 (given by the ratio ${{R}_{P/H}}$ between ${{N}_P}$ and the number of H3K27-marked monomers). As ${{E}_{P - P}}$ increases (Fig. 2b, left), the average number of foci decreases (top panel) with the appearance of larger foci at the expense of smaller ones (middle), while the free nucleoplasmic fraction of PRC1 is strongly reduced (bottom). As ${{E}_{P - H}}$ decreases, foci are in average less numerous and larger and the free nucleoplasmic fraction of PRC1 increased (Supplementary Fig. S2). All these effects are conserved regardless of the values of the other parameters (Supplementary Fig. S2).

Therefore, the observations made by microscopy in SUMO RNAi conditions,i.e. the emergence of fewer but larger foci in a darker nucleoplasmic background (see Fig. 1), are consistent with an increase of the strength of self-attraction ${{E}_{P - P}}$ only (Fig. 2c) but not with a change of ${{E}_{P - H}}$ only (Supplementary Fig. S2). To further validate the model, we investigate the effect of an overexpression of PRC1 by varying ${{R}_{P/H}}\ $(Fig. 2b and c, right panels and Supplementary Fig. S2). We predict an overall increase in the free nucleoplasmic fraction and in the number of condensates that become larger homogeneously, in perfect accordance with what was experimentally observed (Fig. 1). Taken together, polymer simulations indicate that homotypic interactions between PRC1s are necessary to explain the formation of large, intense PRC1 foci observed upon SUMO inactivation, providing indirect evidence that chromatin-mediated phase-separation of PRC1 occurs *in vivo*. Altogether, this suggests that SUMOylation may act as a solubility factor buffering the interactions between PRC1 molecules. Its removal leads to increased PRC1–PRC1 interactions, aberrant PRC1 clustering and the formation of large PRC1 foci.

### Loss of SUMOylation leads to changes in gene expression independent of H3K27me3

**Figure 3.**
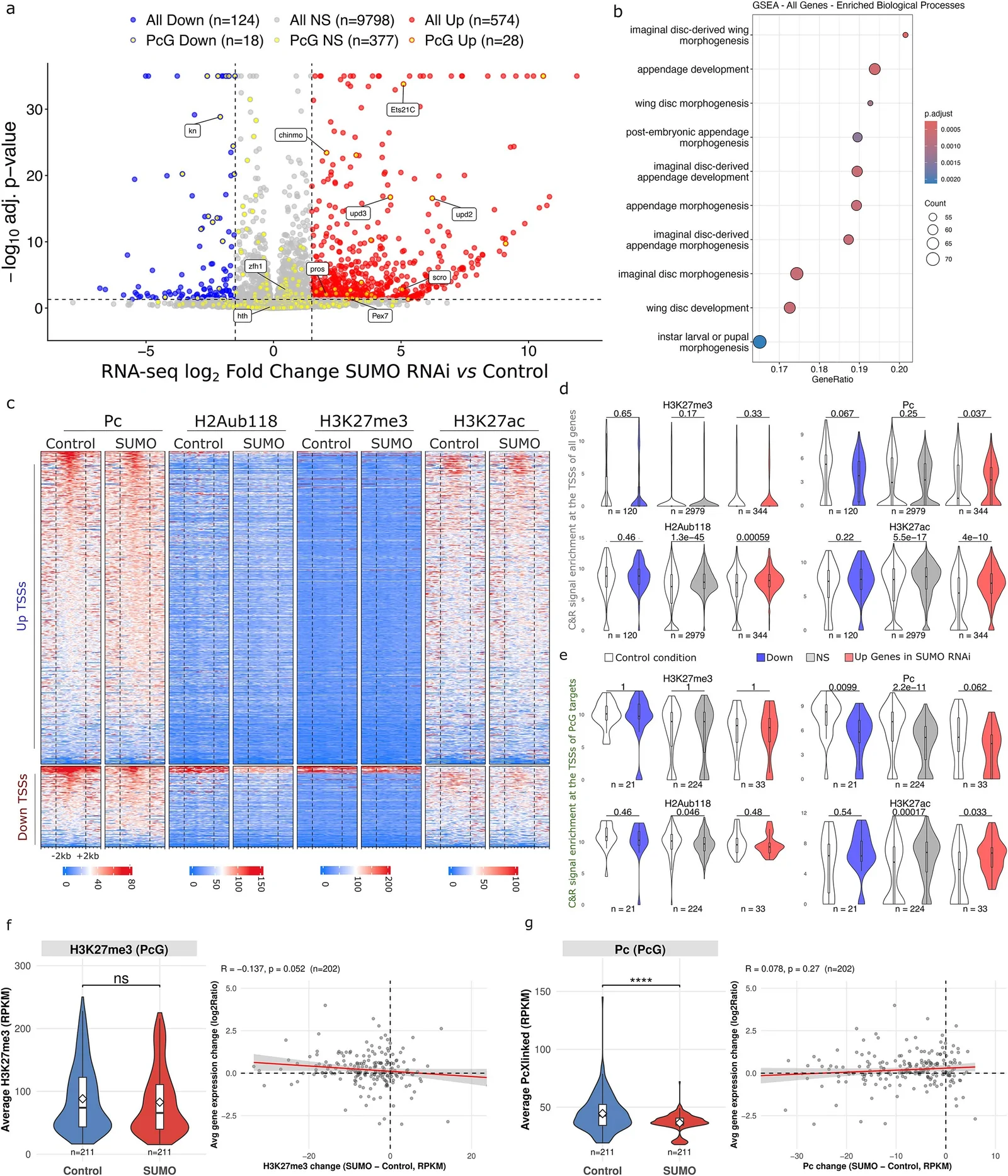
SUMO RNAi leads to gene expression changes independent of H3K27me3 and Pc. (**a**) Volcano plot of the differential gene expression (DE) analysis between SUMO RNAi and Control (reference) sample; (*P*-adjust < .05 and absolute log_2_(Fold-Change) > 1.5). The PcG target genes are highlighted in yellow. The 42 genes with *P*-values ≤ 10^−35^ are capped at -log_10_(*P*-value) = 35 for visualization clarity. (**b**) GSEA of all the DE genes. Top 10 enriched processes are represented. (**c**) Heatmaps of Pc, H2Aub118, H3K27me3, and H3K27ac marks around the transcription start site (TSS) of DEGs, including up-regulated (Up TSSs) and down-regulated (Down TSSs) genes. Comparisons of the normalized C&R signals around the TSSs of all (**d**) and PcG target (**e**) genes between Control and SUMO RNAi. Blue, gray, and red indicate the distributions for the TSSs of the down-, up-, or not significantly deregulated genes in SUMO RNAi compared to Control. The distributions for the Control condition are always shown in white. Reported *P-*values result from comparing the Control and SUMO RNAi distributions using the paired two-sided Wilcoxon statistical test with Benjamini–Hochberg correction for multiple hypothesis testing. The number of points per boxplot (bottom) is reported. (**f, g**) (left panels) Violin plots of the average signal of H3K27me3 (panel f) and Pc (panel g) along the whole PcG TADs in Control and SUMO RNAi conditions. Diamonds indicate the average values. The number of points per boxplot (bottom) is reported. No significant change on the average H3K27me3 was found, whereas Pc is generally reduced. (right panels) Scatter plot of the average H3K27me3 (panel f) and Pc (panel g) marks enrichment versus gene expression change in SUMO RNAi versus Control. Changes (SUMO-Control) on the average H3K27me3 and Pc signal along the whole PcG TADs were not correlated with gene expression (*R* = -0.137, *P* = .052; *R* = 0.078, *P* = .27, respectively). In panels (d), (e),(f), and (g), boxplots show median (central line), Q1 = 25th and Q3 = 75th percentiles (box limits), and Q1 + 1.5 × IQR to Q3 + 1.5 × IQR (whiskers), where IQR is the interquartile range. Outliers are not shown.

Given the dramatic reorganization of PRC1 condensates, we next asked whether SUMO depletion alters gene expression. We performed bulk RNA-seq on SUMO RNAi wing discs (Supplementary Table 1). Because SUMO is required for diverse cellular processes essential for viability [28, 29], we first confirmed that the tissues used were not undergoing widespread apoptosis. RNA levels of canonical apoptotic markers in *Drosophila*, including *rpr, Dronc, Dcp-1, AIF, Alg-2*, and *hid*, showed no significant upregulation (*P*-value < .05 and absolute log_2_(Fold-Change) > 1.5) at the developmental stage analyzed (Supplementary Fig. S3b) [30, 31]. Consistently, SUMO-depleted wing discs appeared overall healthy, although flies with SUMO RNAi either failed to hatch after the pupal stage or emerged with severe wing defects, suggesting that apoptosis might occur later in development. We observed a modest increase (log_2_(Fold-Change) = 0.822) in the pro-apoptotic gene *rpr* [30, 31], which we interpret as reflecting a primed rather than actively dying state, as the tissues remained viable. RNA-seq analysis further revealed diffuse transcriptional changes, with 700 (15%) differentially expressed genes (DEGs), including known Polycomb targets such as *Ets21C, upd1-3*, and *chimno* (Fig. 3a). We did not detect deregulation of the canonical PcG target gene *Ubx* in the wing discs; however, gene set enrichment analysis (GSEA) indicated a coordinated shift toward an active state of cellular growth and developmental reprogramming (Fig. 3b and Supplementary Fig. S3), consistent with a deregulation of the PcG machinery [32].

We next asked whether these expression changes were associated with alterations in chromatin features. To address this point, we performed CUT&RUN profiling for Pc, H3K27me3, H2Aub118, and H3K27ac. Pc occupancy was mildly decreased across the genome, and the Pc signal appeared slightly more diffusely distributed around TSSs in the SUMO-depleted condition (Fig. 3c). This reduction in Pc binding was observed even at PcG target genes that were transcriptionally downregulated (Fig. 3e). On the other hand, the small reduction in Pc occupancy was not accompanied by widespread changes in canonical Polycomb histone modifications. H3K27me3 enrichment was stable genome-wide, with only three novel sites appearing upon SUMO depletion (Fig. 3c and d; Supplementary Fig. S4). PcG target genes also maintained their H3K27me3 signal (Fig. 3e and Supplementary Fig. S5). H2Aub118 showed a slight reduction around differentially expressed PcG target TSSs, but this trend was not statistically significant (*P* = 0.48 Fig. 3d and e). Instead, H2Aub118 levels were reduced near the TSSs of non-differentially expressed PcG genes (*P* = 0.046, Fig. 3d and e), arguing against a role for this modification in DE. As expected, H3K27ac was increased in SUMO RNAi relative to Control at the TSSs of upregulated genes but not on downregulated genes, both genome-wide and at PcG targets (Fig. 3c–e). However, a significant increase was also evident at unchanged genes (Fig. 3d and e). Overall, these changes were small, arguing against a major instructive role for histone modifications in driving the observed changes in gene expression.

Polycomb target genes and H3K27me3-enriched regions form genomic domains. We thus also tested if the average level of H3K27me3 or Pc changed along these domains, and if so, whether they co-occur with gene expression changes (Fig. 3f and g). In line with our differential peak analysis, the overall average levels of H3K27me3 levels did not change along Polycomb domains. Some variations were observable along domains, but these variations did not show a significant correlation with the expression changes of genes located inside these domains (Fig. 3f). Although Pc levels on the Polycomb domains were reduced, the changes on the average Pc level along the individual domains did not show a correlation with the change on gene expression (Fig. 3g).

Together, these results indicate that the transcriptional changes triggered by SUMO depletion, especially down-regulation, do not correlate with modifications of Polycomb-associated histone marks. This suggests that altered PRC1 clustering may contribute to changes in gene expression through mechanisms not directly dependent on H3K27me3.

### Inter-TAD contacts are rewired upon loss of SUMOylation while TAD structures remained stable

The lack of major changes in H3K27me3 and H2Aub118, even at DEGs, suggested that the altered transcription upon SUMO depletion cannot be explained solely by changes in PRC1 levels or by SUMOylation of transcription factors. We reasoned that SUMO RNAi-dependent changes in the nuclear organization of PRC1 might instead alter the higher-order organization of PcG targets and thereby contribute to the observed gene expression changes. PcG domains are known to engage in long-range interactions between repressive TADs, often forming so-called “TAD cliques” that facilitate the coordinated repression of target genes [33, 34]^.^ These PcG-associated TADs are typically enriched for H3K27me3 and depend on PRC1 for their long-range connectivity. We therefore asked whether SUMOylation contributes to 3D chromatin architecture.

**Figure 4.**
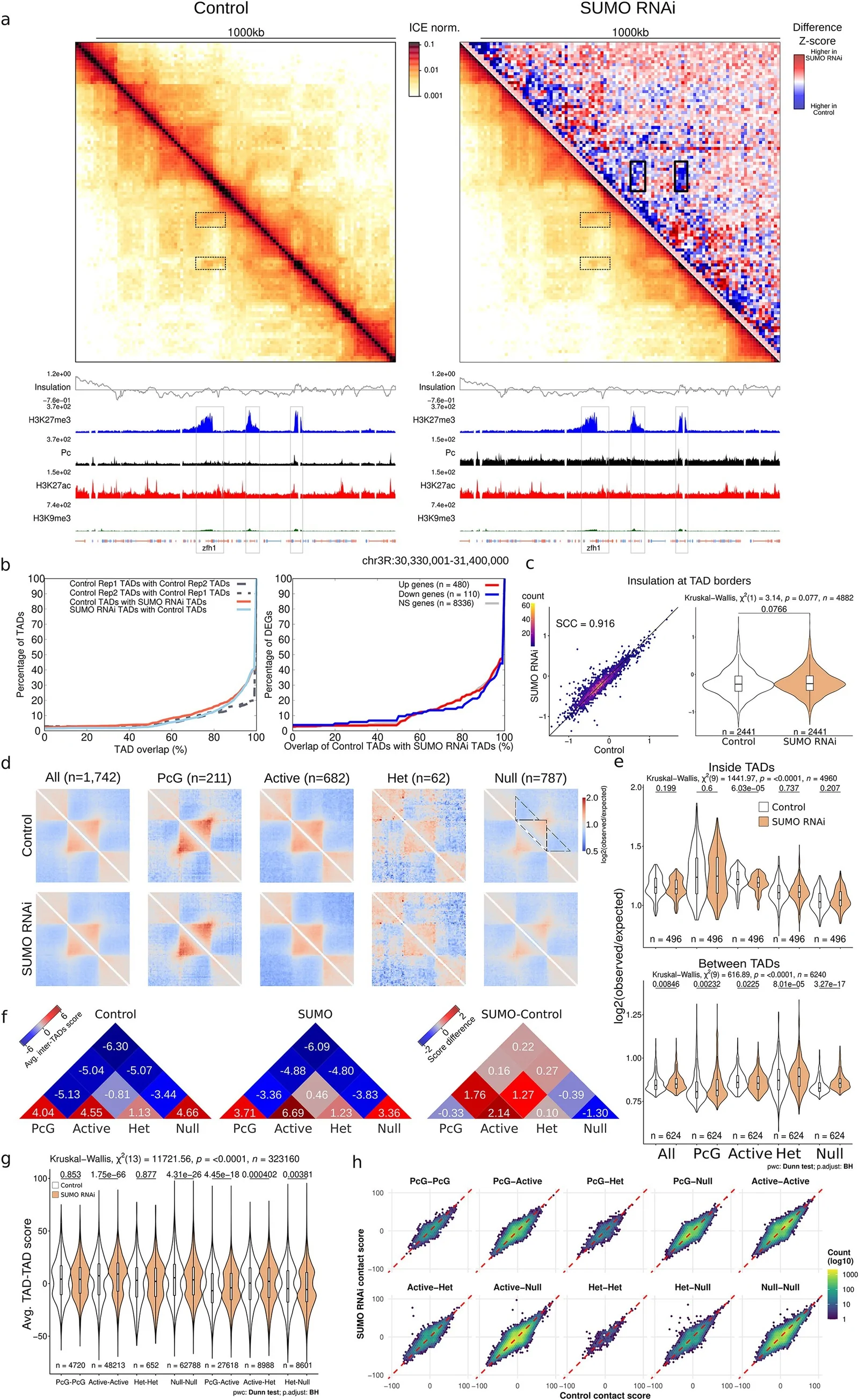
SUMO RNAi leads to genome-wide reorganization of the genome. (**a**) Heatmap of ICE-normalized Hi-C maps at 10 kb resolution in Control and SUMO RNAi samples for the genomic region around the *zfh1* locus (see “Materials and methods” section). C&R tracks for H3K27me3, Pc, H3K27ac, and H3K9me3 marks are shown in the bottom tracks. The rectangles indicate regions of contact differences in PcG–PcG and PcG–Active enriched regions. (**b**) Curves of the percentage of TADs (left) and DEGs (right) as a function of the overlap between TADs in different samples. Comparisons include: Control Rep1 (*n* = 1, 755 TADs) versus Control Rep2 (*n* = 1, 732 TADs), and viceversa, and Control (*n* = 1, 745 TADs) versus SUMO RNAi (*n* = 1, 729 TADs), and viceversa. The curves indicate large overlaps: e.g. 80% of TADs in Control overlap >84% with TADs in SUMO RNAi. Eighty percent of DEGs are hosted in Control TADs with at least 78% of overlap with SUMO RNAi TADs. (**c**) Scatter and violin plots of the insulation score values at the TAD boundaries. Insulation values at Control TAD borders are not significantly different and highly correlated in Control and SUMO RNAi. Boxplots show median (central line), Q1 = 25th and Q3 = 75th percentiles (box limits), and Q1 + 1.5 × IQR to Q3 + 1.5 × IQR (whiskers). Outliers are not shown. *P*-values are shown above each violin plot pair. Statistical comparisons were performed using the Kruskal–Wallis test. (**d**) Aggregate rescaled Hi-C map at 1 kb resolutions at TADs in Control (top) and SUMORNAi (bottom). From left to right, the analysis considers all and chromatin-state-specific TADs. We used ChromHMM [35] to assign chromatin states (colors) to each TAD in the genome (Methods). Chromatin states (colors) include: Active (red) chromatin enriched by H3K27ac, Null (black) chromatin not enriched in any features used for the analysis, HP1 (green) chromatin enriched by H3K9me3, and Polycomb (blue) chromatin enriched by H3K27me3. (**e**) Violin plots show the contact-enrichment values in a central 33 × 33 square (top) and two TAD adjacent reversed triangles of 25 pixels (see the heatmap for Null TADs in Control in the panel c) of the aggregate Hi-C maps presented in panel (c). (**f**) Heatmap of the average inter-TAD score between the four chromatin states in Control and SUMO RNAi samples. The corresponding differential heatmaps (right) clearly indicate an increase of inter-contacts for Active TADs. (**g**) Quantification of the inter- and intra-TAD contact enrichment of each TAD type pair between control and SUMO samples. Boxplots show median (central line), Q1 = 25th and Q3 = 75th percentiles (box limits), and Q1 + 1.5 × IQR to Q3 + 1.5 × IQR (whiskers). Outliers are not shown. *P*-values are shown above each violin plot pair. Statistical comparisons were performed using the Kruskal–Wallis test followed by Dunn’s *post-hoc* pairwise test with Benjamini–Hochberg correction. (**h**) Comparison of contact scores per TAD type, between each TAD pair in Control and SUMO conditions. Each point in the hexbin plots indicates the contact score of each TAD pair in SUMO condition on the *y*-axis and in the Control in the *x*-axis.

To address this, we performed Hi-C experiments to obtain a genome-wide view of chromatin folding in control and SUMO-depleted wing discs. By manual annotation, we noticed some regions enriched in H3K27me3 and Pc that loose contacts in SUMO RNAi compared to Control (Fig. 4a). To systematically identify these differences, we segmented the genome into physical domains (TADs) using TopDom and classified them by histone-mark enrichment using ChromHMM (see “Materials and methods” section, Fig. 4a): Active TADs (H3K27ac-enriched), Polycomb TADs (H3K27me3-enriched), constitutive heterochromatic TADs (H3K9me3-enriched), and null TADs lacking specific marks [8]. We then quantified intra- and inter-TAD contacts in Control and SUMO RNAi samples.

To assess whether TAD organization or insulation changed, we first measured the overlap between TADs called in the Control and SUMO conditions (Fig. 4b and c). As a reference, we compared the overlap of TADs called separately in two Control replicates (gray dashed lines) with the overlap between Control and SUMO conditions (red and blue lines) (Fig. 4b). The TAD partitions of Control and SUMO RNAi data showed strong concordance: over 80% of TADs overlapped by >80%. DEGs were likewise concentrated in highly conserved domains, with 80% of DEGs residing in Control TADs sharing at least 78% overlap with their SUMO RNAi counterparts (Fig. 4b). Insulation scores at Control TAD borders were strongly correlated between conditions (Spearman’s ρ = 0.916) and did not differ significantly (*P* = .077) between the Control and SUMO RNAi Hi-C maps (Fig. 4c). Together, these results indicate that TAD boundaries and insulation are largely preserved upon SUMO depletion, with marginal effects on TAD definition and gene expression.

We next examined aggregated contacts within and between TADs across the different TAD classes (Fig. 4d and e). Intra-TAD contacts were reduced upon SUMO RNAi only within Active TADs, while all other TAD types retained their internal contact strength (Fig. 4e-top). Aggregated contacts in the insulating regions between TADs increased in all TAD categories except from the Active TADs, which showed weakening interactions (Fig. 4e-bottom), but all these changes were marginal. We then extended the analysis from neighbouring TADs to long-range inter-TAD contacts, which revealed a rewiring of inter-TAD interactions upon SUMO depletion (Supplementary Table 2). All TAD classes exhibited increased interactions with active chromatin, including Active–Het and Active–Null contacts (Fig. 4f). In contrast, homotypic interactions of Null chromatin regions were reduced, while PcG–PcG and Het–Het contacts were preserved and, finally, Active–Active interactions became significantly more frequent (Fig. 4g). Importantly, the hexabin plots per category of the inter-TAD contacts in Control versus SUMO RNAi (Fig. 4h) show that these changes were not uniform, with specific contacts that can either increase or decrease. Together, these results indicate that the effect of SUMO depletion extends beyond Polycomb chromatin, inducing ectopic interactions with active chromatin, as seen for PcG–Active domains.

Thus, the deregulation of gene expression in SUMO-depleted wing discs, occurring in the absence of major changes to histone marks, might be associated with the rewiring of TAD–TAD interactions. We thus set out to test whether loss of SUMOylation might rewire PcG–PcG contacts, producing a reconfigured regulatory landscape.

### SUMO KD leads to bidirectional reorganization of PcG–PcG contacts that correlates with PcG target gene expression

**Figure 5.**
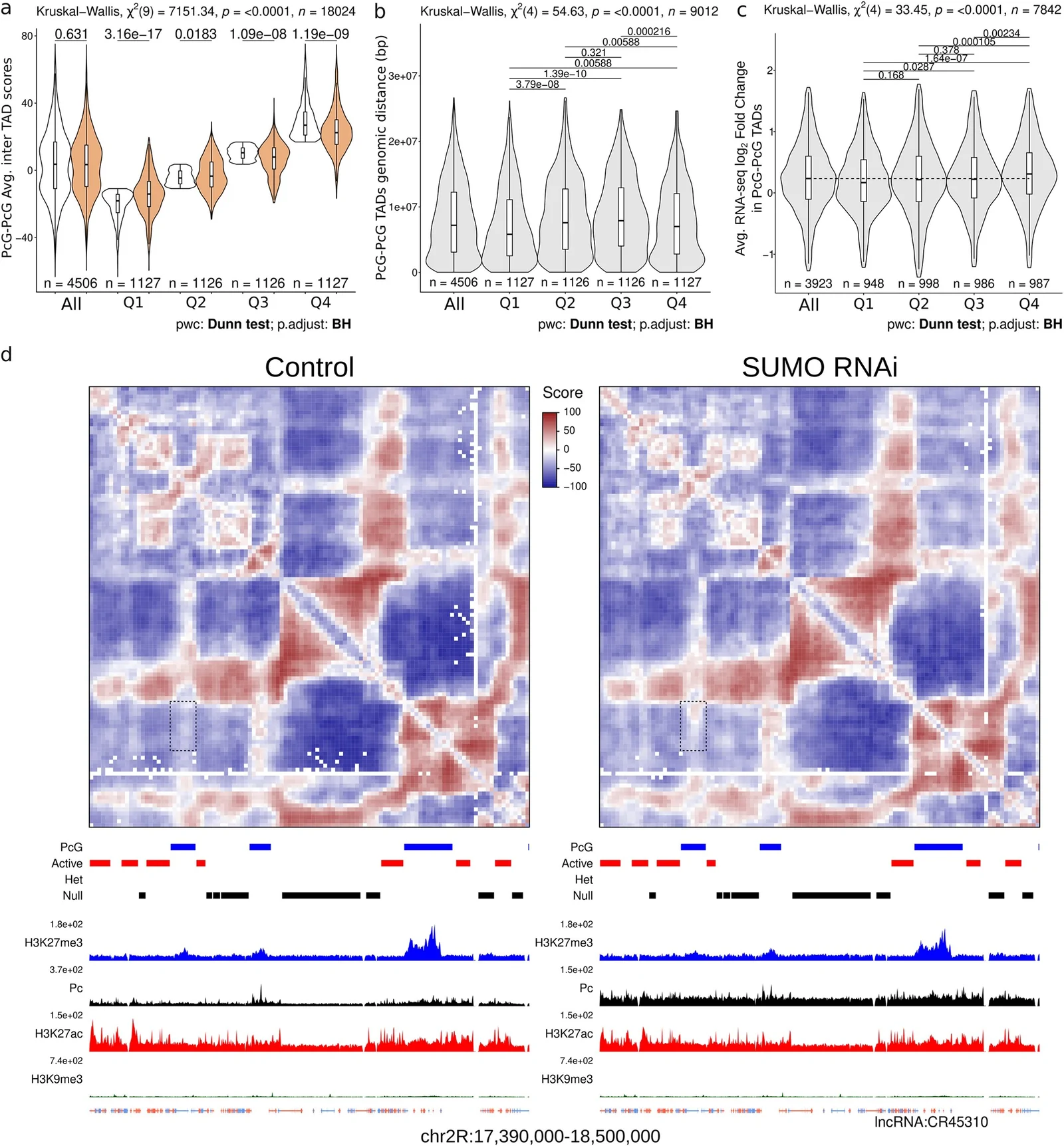
PcG–PcG contact loss at the highest contacting PcG TADs leads to gene de-repression. (**a**) Distribution of the inter-PcG–PcG TAD contact scores. Category All indicates contacts without any filtering. Q1–Q4 indicate the PcG–PcG TAD contacts grouped in quartiles in the Control condition. (**b**) All and quartile-grouped distributions of the TADs’ genomic distance. The genomic distance between TADs is computed between the centres of the domains. (**c**) All and quartile-grouped distributions of average expression log_2_ fold change of TAD pairs. The log_2_ fold change of a TAD is the average log_2_ fold change of the genes assigned to the TAD. A gene is assigned to a TAD if its TSS is contained in the TAD, or, if it is not inside any TAD, to the closest TAD within a 5 kb tolerance. The average log_2_ fold change of a TAD pair is the average log_2_ fold change value of the two TADs involved. (**d**) Example of Hi-C maps for one of the measured contact changes in Q1 that shows an increase of contact score in SUMO RNAi compared to Control. The rectangle indicates the considered contact region. Other examples are provided in Supplementary Fig. S6.

To quantify and characterize the differential inter-TAD chromatin interactions, we analyzed contacts that each TAD makes with other TADs. We first focused on PcG–PcG contacts. To capture the relative changes in PcG chromatin contacts upon SUMO RNAi, we divided PcG–PcG TAD pairs into quartiles based on the strength of contacts they make in Control conditions, and compared their contact scores between Control and SUMO RNAi (Fig. 5a). We considered five categories: the entire distribution of inter-PcG–PcG contacts (All), the pairs that are weakly interacting in Control (Q1, first quartile), the pairs that engage in the strongest interactions in Control (Q4, fourth quartile), and the intermediate quartiles Q2 and Q3. Across the entire distribution, inter-PcG–PcG contacts showed no significant overall change between Control and SUMO RNAi (Fig. 5a, “All,” *P* = .631). However, stratifying by quartile revealed rearrangements: PcG–PcG pairs with the weakest baseline contacts (Q1) gained contact frequency upon SUMO RNAi (*P* = 3.16 × 10^−17^), while pairs with the strongest baseline contacts (Q4) lost contact frequency (*P* = 1.19 × 10^−9^). Q2 and Q3 showed smaller but still significant changes in the same convergent direction (Q2: *P* = .018; Q3: *P* = 1.09 × 10^−8^). This pattern indicates that, rather than a uniform gain or loss of PcG–PcG interactions, SUMO depletion drives a redistribution of PcG–PcG contacts: pairs that normally interact weakly become more connected, while pairs that normally cluster tightly become less so. These two contrasting effects explain why the overall “All” category appears unchanged.

To understand if the changes happened preferentially on the long or short range, we compared the distribution of distances between TAD pair centres across quartiles (Fig. 5b). Although the quartile groups differ in their distance distributions (*P* < .0001, median ∼5–7 Mb across all groups), the differences are not systematically aligned with the magnitude of the contact-score changes observed in panel (a). The contact-score quartile analysis therefore reflects diffuse differences affecting both short- and large-range genomic separations.

We next asked whether the contact rearrangement patterns map onto changes in gene expression. For each PcG–PcG TAD pair, we computed the average log_2_ fold-change of all genes assigned to the two TADs involved (Fig. 5c). The quartile groups differ significantly in their expression-change distributions (*P* < .0001), with Q4 showing the highest median log_2_ fold-change compared to all other quartiles (Q4 versus Q1: *P* = 1.64 × 10^−7^; Q4 versus Q2: *P* = 1.05 × 10^−4^; Q4 versus Q3: *P* = 2.34 × 10^−3^). Thus, the PcG–PcG pairs that lose the strongest contacts upon SUMO RNAi (Q4 in Fig. 5a) are the ones whose genes are most upregulated, consistent with a model in which loss of strong PcG–PcG associations relieves transcriptional repression at PcG target genes.

An example of a region showing increased PcG–PcG contacts in Q1 upon SUMO RNAi is shown in Fig. 5d (chr2R:17 390 000–18 500 000; additional examples in Supplementary Fig. S6). The Hi-C contact maps reveal a weak PcG–PcG interaction (dashed rectangle) that becomes more pronounced in SUMO RNAi compared to Control, which results in down-regulation of CR45310 under the PcG domain. We also note that the region shown in Fig. 4a is in the PcG–PcG contact Q4, and it is an example of lost contacts from the quartile-based analysis.

### PcG–Active TAD interactions have minimal effect on PcG target gene expression

**Figure 6.**
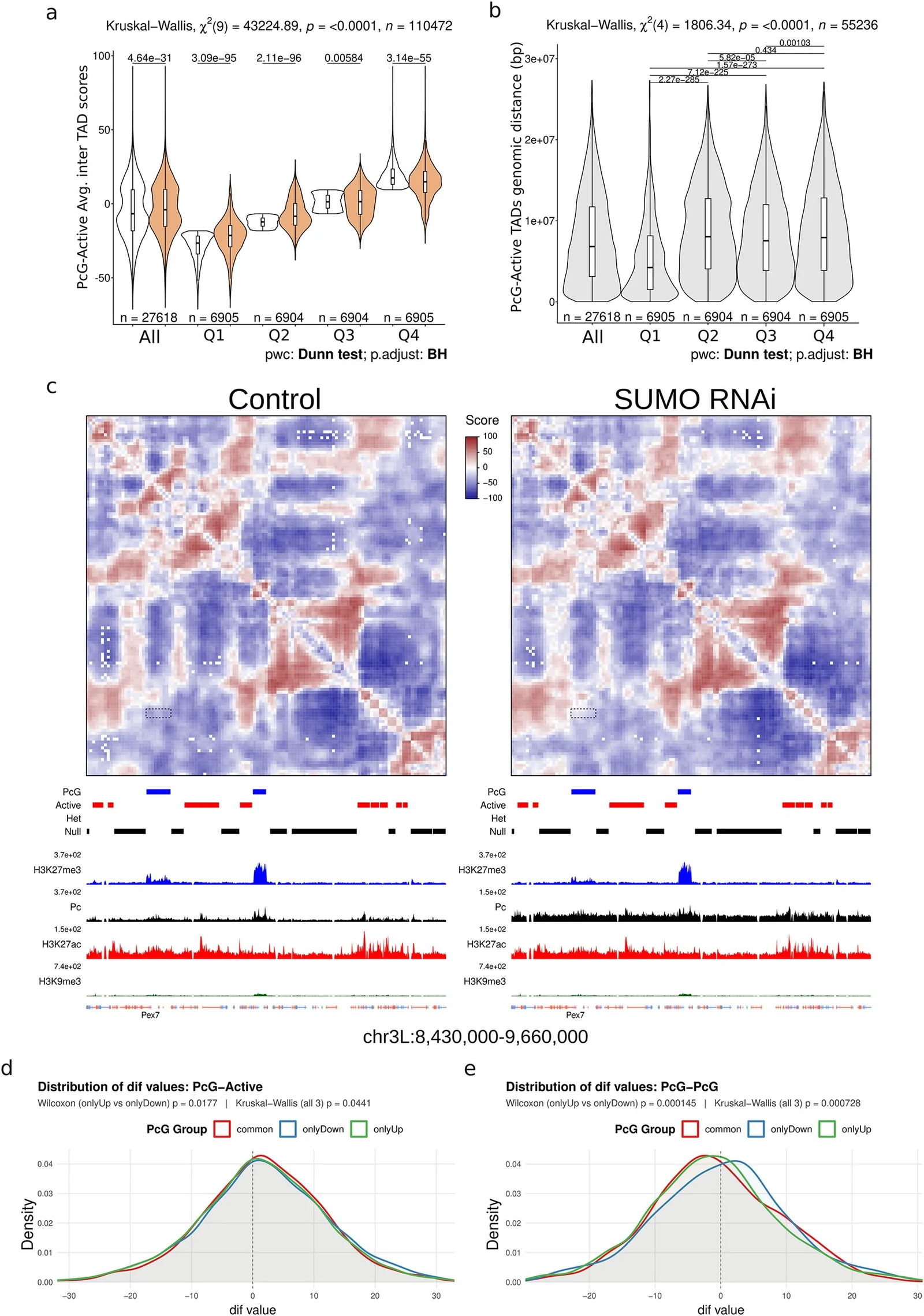
Loss of PcG–PcG is the main driver of gene de-repression. (**a**) Distribution of the inter-PcG–Active TAD contact scores. Category All indicates contacts without any filtering. Q1–Q4 indicate the PcG–Active TADs contacts grouped in quartiles in the Control condition. (**b**) All and quartile-grouped distributions of the genomic distance. (**c**) Example of Hi-C maps for the measured contact changes. The rectangle indicates the contact region taken into consideration. More examples are shown in Supplementary Fig. S7. Density distribution of the contact change values (dif value = contact score SUMO RNAi-contact score Control) between PcG–Active TADs (**d**), and between PcG-PcG TADs (**e**). TADs shown are categorized according to the gene expression change profile: TADs containing only up-regulated genes (onlyUP), TADs containing only down-regulated genes (onlyDown), and TADs containing genes either up or down regulated (common).

The significant overall increase in PcG–Active contacts observed at the genome-wide scale (Fig. 4) prompted us to examine the contact patterns between PcG and Active TADs in more detail. We classified PcG–Active TAD pairs into quartiles based on their contact frequency in Control conditions and compared the Control and SUMO RNAi distributions of contact scores (Fig. 6a). Unlike PcG–PcG contacts (Fig. 5a), the overall distribution of PcG–Active contacts showed a net increase upon SUMO RNAi (All: *P* = 4.6 × 10^−31^). Stratifying by quartile revealed a pattern reminiscent of PcG–PcG contacts: PcG–Active pairs with weak baseline contacts (Q1) gained contact frequency in SUMO RNAi (*P* = 3.1 × 10^−95^), while pairs with the strongest baseline contacts (Q4) lost contact frequency (*P* = 3.1 × 10^−55^). The intermediate quartiles, Q2 and Q3, showed smaller gains. This indicates that PcG–Active contacts undergo an overall shift toward contact gain upon SUMO RNAi, consistent with the broader pattern of de-compartmentalization between PcG and Active chromatin observed at the genome-wide scale.

Examining the genomic-distance distributions of the PcG–Active TAD pair quartiles (Fig. 6b), we found that Q1 pairs with the largest gain in contact frequency sit at shorter linear distances along the chromosome (median ∼5 Mb) than pairs in the higher quartiles (∼7–8 Mb). This indicates that the heterotypic contact gains preferentially occur between PcG and Active TADs that are closer in linear space. An example region (chr3L:8 430 000–9 660 000) illustrating an increase in PcG–Active contact upon SUMO RNAi is shown in Fig. 6c (dashed rectangle); additional examples are provided in Supplementary Fig. S7.

To link structural changes in PcG–Active contacts with transcriptional outcomes, we classified PcG TADs into three groups based on the expression behavior of their resident genes: TADs containing only upregulated genes (onlyUp), only downregulated genes (onlyDown), or both (common). For each group, we computed the distribution of contact-score differences (SUMO − Control) summed across all PcG–Active pairs the TAD participates in (Fig. 6d). The three distributions were essentially overlapping (*P* = 0.044; onlyUp versus onlyDown *P* = 0.0177), indicating that PcG–Active contact changes do not separate upregulated from downregulated PcG TADs in any biologically meaningful way. Notably, contrary to a naive expectation, the onlyDown group showed slightly higher density at the positive end of the distribution, that is, TADs with downregulated genes tended to gain marginally more PcG–Active contacts than TADs with upregulated genes.

By contrast, when we performed the same analysis using PcG–PcG contact differences (Fig. 6e), the three distributions separated clearly: the onlyDown group was shifted toward positive contact-change values (gain of PcG–PcG contacts), while the onlyUp group was shifted toward negative values (loss of PcG–PcG contacts) (*P* = 7.3 × 10^−4^; onlyUp versus onlyDown *P* = 1.5 × 10^−4^). This pattern is consistent with our central finding from Fig. 5: loss of PcG–PcG contacts is associated with gene derepression, while gain of PcG–PcG contacts is associated with downregulation.

To directly compare the explanatory power of each contact type for gene expression change, we plotted the net per-PcG TAD change in contact frequency with the average gene expression change at the same TAD (Supplementary Fig. S8a and b). The net change in PcG–PcG contacts negatively correlates with PcG TAD expression change (Spearman ρ = −0.21, *P* = .003), whereas the net change in PcG–Active contacts shows no significant association (ρ = −0.06, *P* = .38). Consistent with this, when PcG TADs are dichotomized by the direction of their net PcG–PcG contact change, those with net loss of PcG–PcG contacts show significantly greater upregulation than those with net gain (*P* = .02) (Supplementary Fig. S8c).

The apparent paradox in Fig. 6d that onlyDown TADs gain more PcG–Active contacts than onlyUp TADs can be reconciled by considering that PcG–PcG and PcG–Active contact changes co-occur. At the per-TAD level, the net change in PcG–PcG contacts is positively correlated with the net change in PcG–Active contacts (Supplementary Fig. S8d, Spearman ρ = +0.35, *P* = 5 × 10^−7^). In other words, PcG TADs that gain PcG–PcG contacts also tend to gain PcG–Active contacts. This means that the modest gain of PcG–Active contacts seen in the onlyDown group is likely a passive consequence of these TADs also gaining PcG–PcG contacts, rather than a separately regulated event.

Together, these analyses show that although some genes can gain expression upon increased contact with Active TADs, the bulk gain of PcG–Active contacts, while statistically significant, co-occurs with PcG–PcG changes and has no independent association with transcriptional output. This positions the PcG–PcG network as the structural feature most directly tied to PcG-mediated repression.

## Discussion

In this study, we demonstrate that global reduction of SUMOylation profoundly reorganizes the material properties of PRC1 condensates and their associated chromatin domains. Remarkably, these architectural changes and the resulting transcriptional misregulation occur without major alterations in PRC1 binding and H3K27me3 distribution, highlighting higher-order chromatin organization as a separable layer of Polycomb-mediated regulation.

### SUMOylation as a modulator of PRC1 condensate material state

PRC1 functions as a key architectural organizer, forming nuclear foci that facilitate long-range interactions between H3K27me3-enriched domains [1, 6–12]. Our data show that SUMO depletion transforms these foci into enlarged structures with properties consistent with a predominantly gel-like state, exhibiting reduced molecular dynamics and limited internal mixing (Fig. 1e). In our biophysical model, increasing the attraction between PRC1 complexes (E_P–P_) was sufficient to mimic a hypo-SUMOylated state, driving a transition from many small, local clusters to a few substantially larger foci (Fig. 2b). These clusters could act as topological hubs that draw weakly interacting H3K27me3-marked regions closer in physical space (Figs 5 and 6), reshuffling the spatial organization of PcG TADs. This PRC1 reorganization correlates with widespread rewiring of TAD interactions, suggesting that control of PRC1 condensate properties is essential for maintaining the appropriate contact pattern between specific Polycomb domains (Figs 5 and 6).

SUMOylation thus provides a reversible, tunable mechanism to regulate condensate size and material state. While previous work has emphasized protein–protein interactions among PRC1 subunits, or the competitor complexes such as SWI/SNF, in foci formation [10, 13, 26, 36], our findings demonstrate that SUMOylation critically modulates condensate dynamics, and that the formation of aberrant, gel-like condensates upon SUMO loss can drive ectopic 3D TAD interactions that compromise Polycomb domain insulation.

### SUMO gates proper 3D genome organization

We observed that PcG-enriched TADs normally engage in specific long-range interactions, consistent with previous reports [32, 33]. Upon SUMO depletion, these networks are rewired: PcG TADs that normally engage in strong contacts lose those contacts with each other, while gaining ectopic interactions with neighbouring active chromatin (Figs 5 and 6). Although these heterotypic PcG–Active gains were statistically robust at the genome-wide scale, they co-occurred with PcG–PcG changes, which were the contacts most directly associated with transcriptional output (Fig. 6 and Supplementary Fig. S8). This positions the PcG–PcG network as the structural feature most tightly linked to gene misregulation. More broadly, this reorganization suggests that SUMOylation helps constrain PRC1-mediated interactions to appropriate chromatin partners under normal conditions, preventing promiscuous cross-talk between compartments.

Our findings complement prior work showing that chemical disruption of PRC1 condensates abolishes contacts among PcG target regions [12]. Here, SUMO loss drives the opposite extreme of excessive clustering and gel-like properties, which also disrupts proper topology by creating aberrant bridges between normally segregated domains. This biphasic relationship suggests that both too little and too much PRC1 self-association are deleterious, highlighting that both the presence and the proper material state of PRC1 condensates are essential regulators of genome folding.

### Partial mechanistic uncoupling from histone marks

A notable finding is the stability of H3K27me3 despite slightly reduced Pc binding and substantial 3D reorganization of TAD-to-TAD interactions. This demonstrates that H3K27me3 maintenance and higher-order chromatin interactions are mechanistically separable. The persistence of H3K27me3 across most domains, even as their spatial relationships change, suggests that this mark may be more stable than the condensates that organize it.

On the other hand, the increase in H3K27ac, observed upon SUMO depletion mainly at the TSSs of upregulated genes and to a more modest extent at unchanged genes (Fig. 3d–e), points to broader effects on chromatin-modifying activities, either directly or through misregulation of other SUMOylated factors.

### Limitations and future directions

A key consideration is that we employed global SUMO depletion rather than PRC1-specific SUMO mutants. While this approach affects hundreds of SUMOylated proteins, the coherence of the specific changes in PRC1 condensate morphology coupled with specific rewiring of PcG-associated TAD interactions suggests a direct functional link. Supporting this, overexpression of a Pc-3KR mutant (lacking three SUMOylation sites) shows partially enlarged nuclear foci [18]. However, when we replaced the endogenous wild-type Pc gene with a Pc-3KR mutant form by CRISPR-dependent mutagenesis, we failed to detect phenotypic effects on imaginal wing discs or adult flies as strong as in the SUMO RNAi condition, suggesting that other SUMOylation sites in Pc and/or SUMOylation of other PRC1 subunits might compensate for the reduction in Pc SUMOylation. Dissecting the specific SUMOylation sites within PRC1 remains an important aim for future research.

SUMOylation is dynamically regulated during the cell cycle and in response to stress [37–40], suggesting that PRC1 condensate properties may be tuned in specific biological contexts. SUMO can also remodel condensate composition through multivalent interactions with proteins bearing SUMO-interacting motifs (SIMs). These contacts, in turn, recruit SIM-bearing partners, with the balance of SUMO and SIM valency dictating which clients are enriched [41]; within PRC1, the subunit CBX4/Pc2 carries two SIMs that bind SUMO non-covalently and concentrate it at Polycomb foci [42]. Future profiling of the PRC1 interactome under SUMO-depleted conditions might reveal how post-translational modifications reshape condensate composition and function.

### Conclusions and implications

Spatial organization of chromatin, together with its associated proteins, is strongly interlinked with their physical properties [43, 44]. Our findings establish SUMOylation as a critical regulator of PRC1 condensate material properties, 3D genome architecture, and transcriptional fidelity. By showing that aberrant condensate clustering is associated with ectopic inter-TAD contacts and gene misregulation independently of canonical Polycomb-dependent histone marks, we reveal a broader paradigm in which reversible post-translational modifications fine-tune the material state and regulatory capacity of nuclear condensates. This work links phase-separation mechanics directly to chromatin topology and gene expression control, suggesting that post-translational regulation of biomolecular condensation represents a general strategy for coordinating nuclear architecture with transcriptional programs in metazoans. Future studies are needed to explore how fluctuating SUMO levels during development, cell-cycle progression, and stress responses dynamically regulate nuclear condensates to maintain genome organization and function.

## Supplementary Material

gkag851_Supplemental_Files

## Data Availability

The sequencing data generated in this study have been deeposited in the NCBI Gene Expression Omnibus under accession number GSE320399. The description of CUT&RUN, Hi-C, and RNA-seq computational analyses and of the corresponding Figure panels can be found in the Zenodo database at https://doi.org/10.5281/zenodo.21446525.

## Notes

Present address: Dynamics of Biological Systems, Center for Human and Molecular Biology (ZHMB), Saarland University, 66123 Saarbrücken, Germany
