# Supplementary material for: Loss of SUMOylation drives aberrant PRC1 clustering and 3D genome rewiring independent of H3K27me3
Source: Nucleic Acids Res. 2026 Sep 8;54(17):gkag851. doi: 10.1093/nar/gkag851 (PMC13551046; doi:10.1093/nar/gkag851)
Supplement: Supplementary file 1 [file gkag851_supplemental_files.zip › Alilli_Supplementary_Figures_R_07_24.docx]

**Supplementary Figure 1. SUMO RNAi induced on the imaginal wing disc pouch leads to reorganization of PRC1 foci. (a)** Cartoon representing the fly lines used to induce SUMO RNAi on *Drosophila* wing discs. **(b)** Airyscan images of Immuno-staining against Ph, Pc and DAPI staining, in control wing discs. **(c)** Airyscan images of Immuno-staining against Ph, Pc and DAPI staining, in SUMO RNAi wing discs.

**Supplementary Figure 2. Table of simulation results of the biophysical framework. (Columns)** Each column shows simulations of varying strength of steric hindrance ($E_{SH}$). **(Rows)** Each row shows simulations of varying ratio between PRC1 complexes and to H3K27 marked monomer ($R_{P/H}$). **(Heatmaps)** Each heatmap shows simulations of varying strength of PRC1 complexes self-interaction ($E_{P-P}$, horizontal) and of varying PRC1 interaction strength for H3K27-marked monomers ($E_{P-H}$, vertical). **(Blue)** Each blue heatmap shows the number of foci at the end of the simulations. **(Red)** Each red heatmap shows the average volume of foci at the end of the simulations in number for PRC1 complexes. **(Green)** Each green heatmap shows the average PRC1 free nucleoplasmic fraction. The trends shown in **Fig.2b** are not dependent on the values of $E_{SH}$ and $R_{P/H}$. Indeed, an increase of $E_{P-P}$ leads to an in, the number of foci and the PRC1 free nucleoplasmic fraction are decreasing, whereas the volume of foci is increasing.

**Supplementary Figure 3. Gene expression profiles upon SUMO RNAi. (a)** FPKM values of the SUMO gene (*smt3*) in the control and SUMO RNAi samples. p.adjusted= 6.49e-22, n=3. **(b)** Volcano plot showing the DEseq2 results for the apoptotic markers. Significance thresholds are set as: absolute value of the Fold change > 1.5, p.adjusted < 0.05. **(c)** Top 10 enriched GO Molecular Function terms among upregulated (n = 374) and downregulated (n = 87) differentially expressed genes upon SUMO RNAi. Dot color indicates adjusted p-value and dot size indicates GeneRatio. **(d)** Top 6 enriched GO Molecular Function terms among differentially expressed PcG target genes upon SUMO RNAi (upregulated, n = 36; downregulated, n = 16). Dot color indicates adjusted p-value and dot size indicates GeneRatio.

**Supplementary Figure 4.** **SUMO RNAi does not lead to major changes on the distribution of the H3K27me3 mark compared to the control.** **(a)** Scatter plot of the enrichment of H3K27me3 mark, x axis indicates SUMO RNAi and y axis indicates the control. **(b)** Tracks of the differentially bound sites for the H3K27me3 mark in each condition.

**Supplementary Figure 5. Histone marks remain stable around major PcG target genes upon SUMO RNAi. (a)** CUT&RUN signal tracks (RPKM-normalized) for H3K27me3, Pc, H3K27ac, and H3K9me3 at four representative genomic regions containing canonical PcG target genes: the Antennapedia complex (lab, Antp; chr3R:6,640,000-7,090,000), the Bithorax complex (Ubx, Abd-B; chr3R:16,630,000-17,010,000), the engrailed/invected locus (inv, en; chr2R:11,440,000-11,610,000), and the zfh1 locus (chr3R:30,700,000-30,970,000). For each mark, Control and SUMO RNAi tracks are shown, with the maximum y-axis value indicated to the left of each track. H3K27me3 (blue), Pc (black), and H3K27ac (red) profiles are nearly identical between conditions, indicating that the bulk distribution of these histone modifications across PcG domains is largely unaffected by SUMO depletion. H3K9me3 (green) is shown for Control as a reference to indicate that none of the displayed regions is in a heterochromatic state. Gene annotations are shown below each panel; the size scale bar represents 100 kb.

**Supplementary Figure 6.** **SUMO RNAi leads to genome-wide re-organization of PcG-PcG interactions which correlates with changes in gene expression.** Examples of PcG-PcG contact changes upon SUMO RNAi. Hi-C contact-score maps showing two additional genomic regions illustrating PcG-PcG contact changes between Control and SUMO RNAi conditions (related to Fig. 5d). Contact scores are shown on a diverging color scale (red, high; blue, low; the color scale is shown in insets). Dashed rectangles highlight the PcG-PcG contact region under consideration in each panel. Below each pair of Hi-C maps, chromatin-state annotations are shown for PcG (blue), Active (red), Het (green), and Null (black) TADs, followed by RPKM-normalized CUT&RUN tracks for H3K27me3 (blue), Pc (black), H3K27ac (red), and H3K9me3 (green). Gene annotations are displayed at the bottom. **(a)** Region around the Spn43Aa, which is down-regulated, locus (chr2R:6,940,000-7,650,000), showing a PcG-PcG contact that increases in SUMO RNAi. **(b)** Region around the chinmo, which is up-regulated, locus (chr2L:280,000-1,880,000), showing a PcG-PcG contact that decreases in SUMO RNAi. Together with the example shown in Fig. 5d, these regions illustrate the heterogeneity of PcG-PcG contact remodeling observed upon SUMO depletion.

**Supplementary Figure 7.** **SUMO RNAi leads to genome-wide re-organization of PcG-Active interactions.** Additional examples of PcG-Active contact changes upon SUMO RNA. Hi-C contact-score maps showing two additional genomic regions illustrating PcG-Active contact changes between Control and SUMO RNAi conditions (related to Fig. 5d). Contact scores are shown on a diverging color scale (red, high; blue, low; the color scale is shown in insets). Dashed rectangles highlight the PcG-PcG contact region under consideration in each panel. Below each pair of Hi-C maps, chromatin-state annotations are shown for PcG (blue), Active (red), Het (green), and Null (black) TADs, followed by RPKM-normalized CUT&RUN tracks for H3K27me3 (blue), Pc (black), H3K27ac (red), and H3K9me3 (green). Gene annotations are displayed at the bottom. **(a)** Region around the chr3L:10,690,000-12,250,000, showing a PcG-Active contact that increases in SUMO RNAi. This interaction did not lead to a gene expression change. **(b)** Region around the CG17777, which is up-regulated, locus (chrX:1,940,000-2,640,00), showing a PcG-Active contact that decreases in SUMO RNAi. Together with the example shown in Fig. 5d, these regions illustrate the heterogeneity of PcG-PcG contact remodeling observed upon SUMO depletion.

**Supplementary Figure 8. The loss of PcG-PcG contacts, but not the gain of PcG-Active contacts, correlates with derepression of PcG target genes upon SUMO RNAi.** For each PcG TAD (one point per TAD), the net change in contact score (SUMO − Control) was computed as the sum of contact-score differences across all pairs the TAD participates in, separately for PcG-PcG and PcG-Active partner types. The TAD's average gene expression change was computed as the mean log2 fold-change of all genes assigned to it. **(a)** Net PcG-PcG contact change versus PcG TAD gene expression change. PcG TADs that lose PcG-PcG contacts show stronger upregulation of their resident genes (Spearman rho = −0.208, p = 0.003, n = 211). **(b)** Net PcG-Active contact change versus PcG TAD gene expression change. No significant correlation is observed (Spearman rho = −0.062, p = 0.382, n = 211), indicating that gain of PcG-Active contacts is not associated with transcriptional changes at PcG target genes. **(c)** Boxplot comparing PcG TAD gene expression change between TADs with net loss versus net gain of PcG-PcG contacts. TADs with net loss of PcG-PcG contacts show significantly higher upregulation than those with net gain (Wilcoxon p = 0.020). Boxplot center lines indicate medians; box limits show the 25th and 75th percentiles; whiskers extend to 1.5 × interquartile range. **(d)** Net PcG-PcG contact change versus net PcG-Active contact change. The two contact-change types are positively correlated at the per-TAD level (Spearman rho = 0.347, p = 5.05 × 10⁻⁷, n = 211), indicating that PcG TADs which gain PcG-PcG contacts also tend to gain PcG-Active contacts and vice versa. This co-occurrence accounts for the apparent paradox in Fig. 6d, where the onlyDown TAD group shows slightly higher PcG-Active contact gains than the onlyUp group, despite PcG-Active gain itself being uncorrelated with expression change (panel b). In all scatter plots, lines represent linear regression fits with 95% confidence intervals shown in grey; dashed reference lines indicate zero. Spearman rank correlation coefficients and p-values are shown in each panel.
